# *Dracaena cochinchinensis* stemwood extracts inhibit amyloid-β fibril formation and promote neuronal cell differentiation

**DOI:** 10.3389/fphar.2022.943638

**Published:** 2022-09-06

**Authors:** Dusadee Ospondpant, Xiong Gao, Tina Tingxia Dong, Karl Wah Keung Tsim

**Affiliations:** ^1^ Division of Life Science, Center for Chinese Medicine and State Key Laboratory of Molecular Neuroscience, The Hong Kong University of Science and Technology, Hong Kong, Hong Kong SAR, China; ^2^ Shenzhen Key Laboratory of Edible and Medicinal Bioresources, HKUST Shenzhen Research Institute, Shenzhen, China

**Keywords:** Alzheimer’s disease, Aβ aggregation, neuroprotection, neurite outgrowth, *Dracaena cochinchinensis*

## Abstract

Alzheimer’s disease (AD) is a progressive neurodegenerative disorder characterized by the deposition of amyloid plaques in the brain. The prevention of amyloid-β (Aβ)-induced neuronal toxicity is considered a major target for drug development for AD treatment. *Dracaena cochinchinensis* (Lour.) S.C. Chen, a Thai folk medicine named “Chan-Daeng,” is a member of the Asparagaceae family. The stemwood of *D. cochinchinensis* has been traditionally used for its antipyretic, pain relief, and anti-inflammatory effects. The aim of the present study was to determine the pharmacological activities of ethanol and water extracts of *D. cochinchinensis* stemwood in blocking the Aβ fibril formation, preventing Aβ-mediated cell toxicity, and promoting neuronal differentiation in cultured PC12 cells. The herbal extracts of *D. cochinchinensis* stemwood prevented the formation of Aβ fibrils and disassembled the aggregated Aβ in a dose-dependent manner. Additionally, they prevented Aβ fibril-mediated cell death. The synergy of the herbal extract with a low dose of the nerve growth factor showed an increase in the protein expression of neurofilaments, that is, NF68, NF160, and NF200. These findings suggest that the extracts of *D. cochinchinensis* stemwood may be used for AD treatment by targeting Aβ fibril formation and inducing neuron regeneration.

## Introduction

Alzheimer’s disease (AD) is the most common form of dementia and causes a heavy burden on patients and their families (World Health Organization, 2021). It is a progressive neurodegenerative disorder initiated several years before the development of symptoms such as loss of memory, communicating problems, and behavioral changes (Alzheimer´s Association, 2021). Currently, there is no cure for AD. However, preventing or attenuating AD progression may minimize the disease burden. The US Food and Drug Administration has approved five drugs for AD treatment, including acetylcholinesterase inhibitors, N-methyl-D-aspartate receptor antagonists, and anti-amyloid antibodies. Although these drugs are used for AD treatment, they cannot prevent neuronal loss during AD progression or cure the disease (U.S. Food and Drug Administration, 2021). The cause of AD is not yet fully understood. Senile plaques composed of amyloid-β (Aβ) aggregates and fibrils are pathological hallmarks of AD. Various lines of evidence support the amyloid hypothesis, which is also a target for the development of AD drugs (Hardy and Selkoe, 2002; Chen et al., 2017).

Aβ accumulation may be related to neuronal damage and synaptic dysfunction, leading to mild cognitive impairment and severe memory loss (Näslund et al., 2000; Tsai et al., 2004). Neuronal development and regeneration, as reflected by neurite outgrowth or neuronal differentiation, during neurological disorders may be considered as therapeutic interventions for AD. Neurite outgrowth is typically induced by neurotrophic factors, including the nerve growth factor (NGF) (Calabrese, 2008). However, the reduction of NGF in the brain occurs during aging and in neurodegenerative diseases (Bartus, 2000; Budni et al., 2015). Unfortunately, NGF cannot pass through the blood–brain barrier (BBB), leading to difficulties in its therapeutic development (Granholm et al., 1998). Searching for novel small molecules that inhibit Aβ aggregation and promote neurite outgrowth may be an advantageous approach for AD treatment.

Medicinal plants have received significant scientific and commercial attention. Many of them have shown efficacy in treating AD and/or promoting neurite outgrowth (Kumar and Nisha, 2014; Xu et al., 2019; Hu S. L. et al., 2020). In Asian historical records, use of herbal medicine has been a common practice for several years. Thai herbal medicine has been used for over a few hundred years in clinical practice. In Thailand, the stemwood of *Dracaena cochinchinensis* (Lour.) S.C. Chen, also named *Dracaena loureiri* Gagnep, is a folk medicine called “Chan-Daeng,” which is a member of the Asparagaceae family. This medicinal herb has traditional applications as an antipyretic, anti-inflammatory, and in pain relief (Thai Traditional Medicine Association, 1964; Reanmongkol et al., 2003). Additionally, there are many herbal formulas in Thai folk medicine containing *D. cochinchinensis* stemwood for curing a variety of medical problems. As recorded in the Royal Medical Book of King Rama V in AD 1870, the stemwood of *D. cochinchinensis* is prescribed for heart disorders causing irritability, touchiness, and short temper (Committee on Document and Archive Preparation, 1999). These traditional signs of illness may rely on AD symptoms (Mulholland, 1979). Here, the role of *D. cochinchinensis* stemwood in blocking Aβ fibril formation and preventing Aβ fibril-induced toxicity was determined. Simultaneously, the stimulation of neuronal differentiation triggered by the herbal extract was examined in cultured PC12 cells. The stemwood of *D. cochinchinensis* is proposed as an alternative remedy for the treatment of AD.

## Materials and methods

### Chemicals

Resveratrol, loureirin A, loureirin B, and pterostilbene (≥98% purity, as determined by high-performance liquid chromatography (HPLC)) were purchased from Chengdu Must Bio-Technology Co., Ltd. (Chengdu, China). Dimethyl sulfoxide, 1,1,1,3,3,3,-hexafluoro-2-propanal, thioflavin-T (ThT), and 3-(4,5-dimethylthiazol)-2,5-diphenyl-tetrazolium bromide (MTT) were purchased from Sigma-Aldrich (St. Louis, MO, United States).

### Preparation of extracts from the stemwood of *D. cochinchinensis*

The stemwood of *D. cochinchinensis* was obtained from an herbal drugstore in Krung Thep Maha Nakhon (Bangkok, Thailand). The coarse powder of *D. cochinchinensis* (20 g) was sonicated in 0.4 L of 90% ethanol, 50% ethanol, or water for 20 min and re-sonicated twice before filtering, rotary evaporating, and freeze-drying to obtain the extracts. DCS_EtOH90_, DCS_EtOH50,_ and DCS_water_ were named as the *D. cochinchinensis* stemwood (DCS) extracts from 90% ethanol, 50% ethanol, and water, respectively.

### HPLC analysis of the *D. cochinchinensis* stemwood extracts

The *D. cochinchinensis* extracts (10 mg), that are, DCS_EtOH90_, DCS_EtOH50_, and DCS_water_, were dissolved in ethanol and analyzed by HPLC-DAD with a TC-C18 column (4.6 × 250 mm, 5 μm) (Agilent, Santa Clara, CA, United States). The mobile phase was 0.2 % formic acid solution (A) with acetonitrile (B) using a gradient program of approximately 10 %–15 % (B) at 0–10 min, 15 %–25 % (B) at 10–30 min, 25 %–35 % (B) at 30–55 min, 35 %–40 % (B) at 55–65 min, 40 %–60 % (B) at 65–90 min, and 60 %–100 % (B) at 90–96 min. The injection volume was 5 μl. The column temperature was 30°C and the absorbance was measured at 300 nm.

### Preparation of Aβ_1-42_ fibrils

Purified synthetic Aβ_1-42_ (GL Biochem, Shanghai, China) was dissolved in 100% hexafluoroisopropanol and sonicated for 20 min at 25°C. The Aβ_1-42_ monomer solution at a stock concentration of 1 mM was aliquoted and dried in a fume hood overnight for complete removal of hexafluoroisopropanol and then stored at −20°C. For Aβ_1-42_ fibril formation, the dried Aβ_1-42_ peptide film was resuspended in 20 μl dimethyl sulfoxide, dissolved in 10 mM HCl, diluted to a final concentration of 100 μM, and vortexed for 1 min (Stine et al., 2011). The solution of the Aβ_1-42_ peptide was incubated at 37°C for 6 days to form fibrils before further analysis.

### ThT fluorescent assay

The amount of Aβ_1-42_ fibrils was determined using the ThT assay. The intensity of the ThT fluorescent dye was used to quantify the Aβ_1-42_ fibrils (Biancalana and Koide, 2010). To inhibit Aβ fibril formation, the Aβ_1-42_ monomer (10 µM) was subjected to aggregation or co-aggregation with various concentrations of herbal extracts and incubated at 37°C for 6 days. In the disassembly of Aβ aggregation, Aβ fibrils (10 µM) aged for 6 days were incubated with or without herbal extracts at 37°C for 5 days. A ThT fluorescent dye was added at a final concentration of 20 µM. ThT fluorescence was measured at the excitation and emission wavelengths of 435 and 488 nm, respectively, in a 96-well black plate. The fluorescence images of ThT-stained Aβ_1-42_ fibrils were obtained using an SP8 confocal microscope (Leica Microsystems, Germany) with a ×63 oil immersion objective at a wavelength of 405 nm.

### Atomic force microscopy assay

The morphology of Aβ1−42 fibrils or aggregates was observed in the tapping mode using an atomic force microscope (AFM) coupled with a Nanoscope V controller (Veeco, Tokyo, Japan) and a probe with a tip diameter <10 nm. After 6 days of Aβ1−42 monomers and herbal extract incubation or after 5 days of Aβ fibril and herbal extract co-incubation, the sample (30 µl) was collected and applied to mica sheets (1 × 1 cm^2^). The AFM scan size was used to detect Aβ_1−42_ fibrils or aggregates at 2.5 and 10 μm, and 2.5 and 20 μm, respectively.

### Cell culture

Rat pheochromocytoma PC12 cells derived from the rat adrenal medulla were obtained from the American Type Culture Collection (ATCC 1721, Manassas, VA, United States). PC12 cells were cultured in DMEM supplemented with 6% fetal bovine serum (FBS), 6% horse serum, and 1% penicillin/streptomycin (10,000 U/mL and 10,000 μg/ml). Mouse neuroblastoma and rat glioma NG108-15 cells were cultured in DMEM containing 10% FBS and 1% penicillin/streptomycin. Mouse microglial BV2 cells were cultured in DMEM supplemented with 10% heat-inactivated FBS and 1% penicillin/streptomycin. The cells were maintained in a humidified incubator with 5% CO_2_ at 37°C and sub-cultured until passage 20.

### Cell viability assay

Cell viability was assessed using the MTT assay. Cells were seeded in a 96-well plate at a density of 1 × 10^4^ cells/well and cultured for 24 h. PC12 cells were serum-starved (1% FBS and 1% horse serum) for 24 h. To examine the preventive role of the *D. cochinchinensis* extract in Aβ_1-42_ fibril-induced cell death, cells were treated with 6 days of co-aggregated Aβ_1-42_ monomer (10, 15, and 20 µM) and *D. cochinchinensis* extracts for 24 h. In determining the protective role of *D. cochinchinensis* extract in Aβ_1-42_ fibril-induced cell toxicity, PC12, and NG108-15 cells were pre-treated with the extracts for 24 h and then exposed to Aβ_1-42_ fibrils at 10 or 20 µM for 24 h. BV2 cells were pre-treated with extracts for 4 h and then treated with 15 μM Aβ_1-42_ fibrils for 20 h. The MTT solution was added at a final concentration of 0.5 mg/ml for 2 h. The purple formazan in viable cells was dissolved in dimethyl sulfoxide, and the absorbance was measured at 570 nm.

### Neurite outgrowth assay

Cell differentiation was determined by the length of the neurites, that is, a differentiated cell should have a neurite longer than the diameter of the cell body. The cells were seeded at low cell density in a 6-well plate (2 × 10^4^ cells/well) and cultured for 24 h. After 24 h of serum starvation, the cells were exposed to the *D. cochinchinensis* extracts with or without a low concentration of NGF (1.5 ng/ml) for 48 h. NGF at 50 ng/ml was used as a positive control. The morphology of PC12 cells was observed under a phase-contrast microscope (Carl Zeiss, Oberkochen, Germany), ×10 objective, and photographed with a digital camera (SPOT basic software, Diagnostic Instruments, MI). The neurite length of approximately 100 cells, counted from five randomly chosen visual fields, was quantified using the ImageJ software, according to the neurite length at < 15 μm, 15–30 μm, and >30 μm.

### DNA transfection and luciferase assay

PC12 cells (5 × 10^4^ cells/well) were cultured in a 24-well plate for 24 h and transiently transfected with cDNA constructs of pNF68-Luc or pNF200-Luc (Xu et al., 2019) using the jetPRIME reagent (Polyplus Transfection, NY, United States). After 4 h of transfection, the cells were serum-starved for 24 h and treated with herbal extracts with or without 1.5 ng/ml NGF for 24 h. For the luciferase activity assay, the cells were lysed with 100 mM potassium phosphate buffer (pH 7.8) containing 0.2% Triton X-100 and 1 mM dithiothreitol, followed by agitation and centrifugation at 4°C. The supernatant was collected and the luciferase activity was measured using the Luciferase Assay System kit (Promega, Madison, WI, United States). The luminescence was quantified using a GloMax^®^ 96 Microplate Luminometer and normalized to the amount of protein using the Bradford method with a kit from Bio-Rad (Hercules, CA, United States).

### Western blot assay

Protein quantification was performed by Western blotting. Aβ_1-42_ after 1 and 6 days of incubation were collected and separated on 10% sodium dodecyl sulfate-polyacrylamide gels. PC12 cells at 1 × 10^5^ cells/well in a 12-well plate were serum-starved for 24 h and treated with herbal extracts in the presence or absence of 1.5 ng/ml NGF for 48 h. NGF (50 ng/ml) served as a positive control. The cells were lysed with a low-salt lysis buffer (150 mM NaCl, 10 mM HEPES, 1 mM EDTA, 1 mM EGTA, 1% NP-40, 0.01% sodium dodecyl sulfate, and 0.1 M Tris-HCl, pH 7.6) containing protease inhibitors, that is, aprotinin, leupeptin, benzamidine, and pepstatin A. The cell lysates were centrifuged at 12,000 rpm for 15 min at 4°C. Protein concentration was measured using the Bradford assay. Proteins (20 µg) were separated on 8% sodium dodecyl sulfate-polyacrylamide gels and transferred to nitrocellulose membranes. The membranes were blocked with 5% non-fat milk and probed with antibodies against amyloid-fibril OC (Millipore, Bedford, MA, United States), neurofilament-68 (NF68), neurofilament-160 (NF160), neurofilament-200 (NF200), and α-tubulin (Cell Signaling Technology, Danvers, MA, United States) overnight at 4°C. Membranes were subsequently incubated with horseradish peroxidase-conjugated secondary antibody for 1 h at room temperature and developed using an enhanced chemiluminescence Western blotting detection kit (Thermo Fisher Scientific, Waltham, MA, United States). The protein bands were photographed using the ChemiDoc Touch Imaging System (Bio-Rad), and the band intensities were analyzed using the ImageJ software.

### Statistical analysis

Data were acquired from at least four independent experiments and are presented as mean ± SEM. Statistical tests were performed using one-way analysis of variance with Tukey’s post-hoc test and GraphPad Prism 9.0.0. Statistical significance is indicated by * *p*-value < 0.05, ** *p*-value < 0.01, and *** *p*-value < 0.001.

## Results

### Extracts of *D. cochinchinensis* stemwood suppress Aβ_1-42_ fibril formation

**FIGURE 1:**
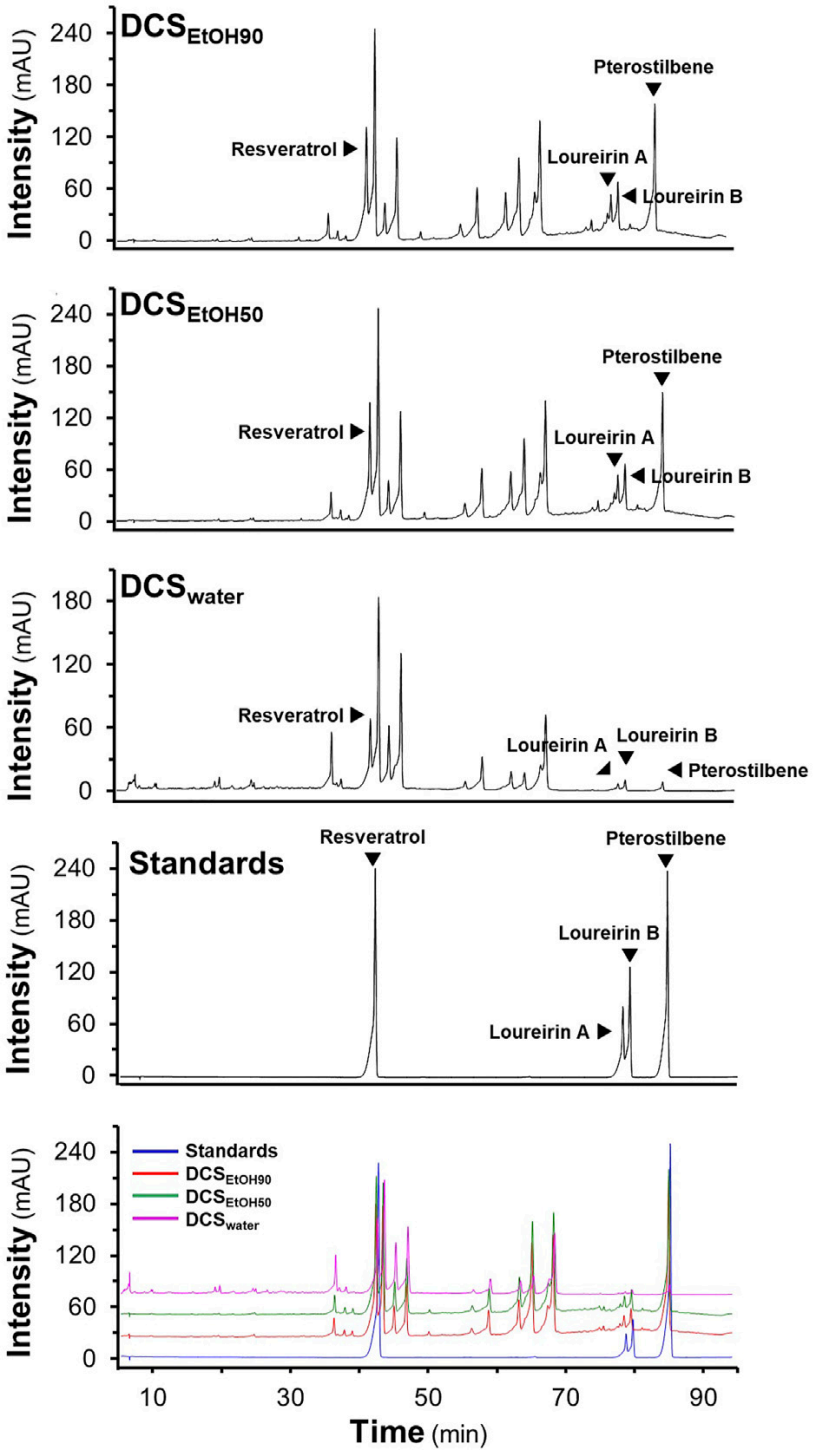
HPLC fingerprints of *D. cochinchinensis* stemwood extracts. The stemwood of *D. cochinchinensis* (10 mg/ml each) extracted from 90% ethanol (DCS_EtOH90_), 50% ethanol (DCS_EtOH50_), and water (DCS_water_), and standard compounds (1 mM) were analyzed by HPLC with a DAD detector at the absorbance of 300 nm, with a volume injection of 5 μl. The characterization of peaks in HPLC chromatograms was mainly identified by the migration of the reference compounds (resveratrol, loureirin A, loureirin B, and pterostilbene).

The stemwood of *D. cochinchinensis* extracted from 90% ethanol, 50% ethanol, and water was named DCS_EtOH90_, DCS_EtOH50,_ and DCS_water_, respectively. The extraction yields were approximately 16% for DCS_EtOH90_, 17% for DCS_EtOH50_, and 2.4% for DCS_water_ (Supplementary Table S1). HPLC chromatograms of the extracts were obtained. Based on their migration time with the known chemicals, four chemicals of *D. cochinchinensis* extracts were identified, that is, resveratrol, loureirin A, loureirin B, and pterostilbene (Figure 1). DCS_EtOH90_ and DCS_EtOH50_ showed abundant peaks of resveratrol, loureirin A, loureirin B, and pterostilbene compared with DCS_water_. In line with the extractive efficacy, DCS_EtOH90_ exhibited more absorbance peaks. The amounts of phytochemicals in the different extracts were measured using HPLC, as shown in Supplementary Table S1. The amounts of resveratrol, loureirin A, loureirin B, and pterostilbene in DCS_EtOH90_ and DCS_EtOH50_ were similar, ranging from 11 to 15 μg/mg in the dried extracts. However, these chemicals were much lower in DCS_water_, ranging from 0.8 to 8 μg/mg.

**FIGURE 2:**
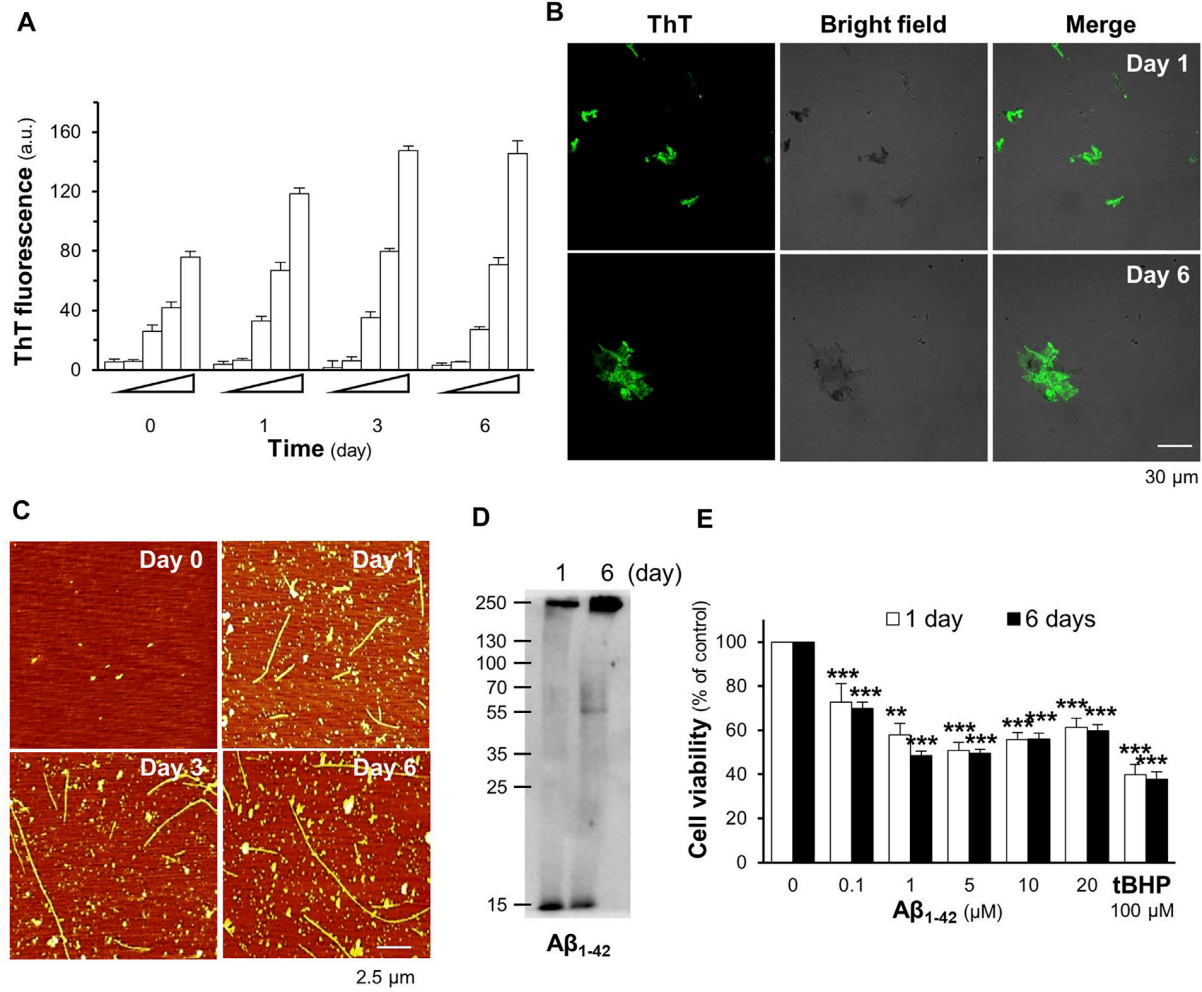
Aβ_1-42_ fibrils induce cell death in PC12 cells. **(A)** The aggregation of the Aβ_1-42_ monomer (0.1–20 μM) incubated at 37°C for 0, 1, 3, and 6 days was analyzed using the ThT fluorescent assay. **(B)** The fluorescence images of ThT-stained Aβ aggregates (10 μM) at 1 and 6 days of aging **(C)** The Aβ_1-42_ monomer at 10 μM aged at 37°C for 0, 1, 3, and 6 days was mounted for the AFM analysis. The formation of Aβ_1-42_ fibrils was observed at a scan size of 2.5 μm. **(D)** Western blotting of Aβ_1-42_ monomer (approximately 15 kDa) and aggregates (>55 kDa). **(E)** Aβ_1-42_ (0.1–20 μM) aggregates at days 1 and 6 were applied to PC12 cells for 24 h. The MTT assay was used as an indicator of cell viability. Graphs represent the mean ± SEM for n ≥ 4. The significant difference between untreated and treated cells with Aβ_1-42_ aggregates is represented by ***p < 0.001.

The formation of Aβ_1-42_ fibrils and their toxicity in cultured cells were elucidated. In the ThT fluorescent assay, the aggregation of Aβ_1-42_ increased in a time- and concentration-dependent manner (Figure 2A). Aβ_1-42_ fibril formation at concentrations of 5–20 µM inclined steadily toward day 3 and plateaued on day 6 of incubation (Figure 2A). Fluorescent images of the Aβ aggregates at 10 µM were detected in a dish after staining with ThT dye at the first and sixth days of aging, as shown in Figure 2B. AFM images of 10 μM Aβ_1-42_ incubated for 1 day showed protofibrils and fibrils; however, the formation of long and straight fibrils was revealed on day 6 of aging (Figure 2C). The aggregation of Aβ_1-42_ was further validated by Western blotting, which showed an increase in the aggregated form of Aβ_1-42_ at >55 kDa after 6 days of aging (Figure 2D). To determine Aβ_1-42_-induced cell death, treatment with Aβ_1-42_ aggregates at different concentrations, ranging from 0.1 to 20 μM, for 24 h decreased cell viability in a dose-dependent manner (Figure 2E), was carried out. In cultured PC12 cells, maximal cell death was observed at approximately 1 µM and beyond. Cell death induced by Aβ aggregates was observed in other brain-derived cells, specifically BV2 cells and NG108-15 cells (Supplementary Figure S1A). Based on these results, the fibril formation of 10 μM Aβ_1-42,_ incubated for 6 days, was chosen to induce cytotoxicity in subsequent experiments.

**FIGURE 3:**
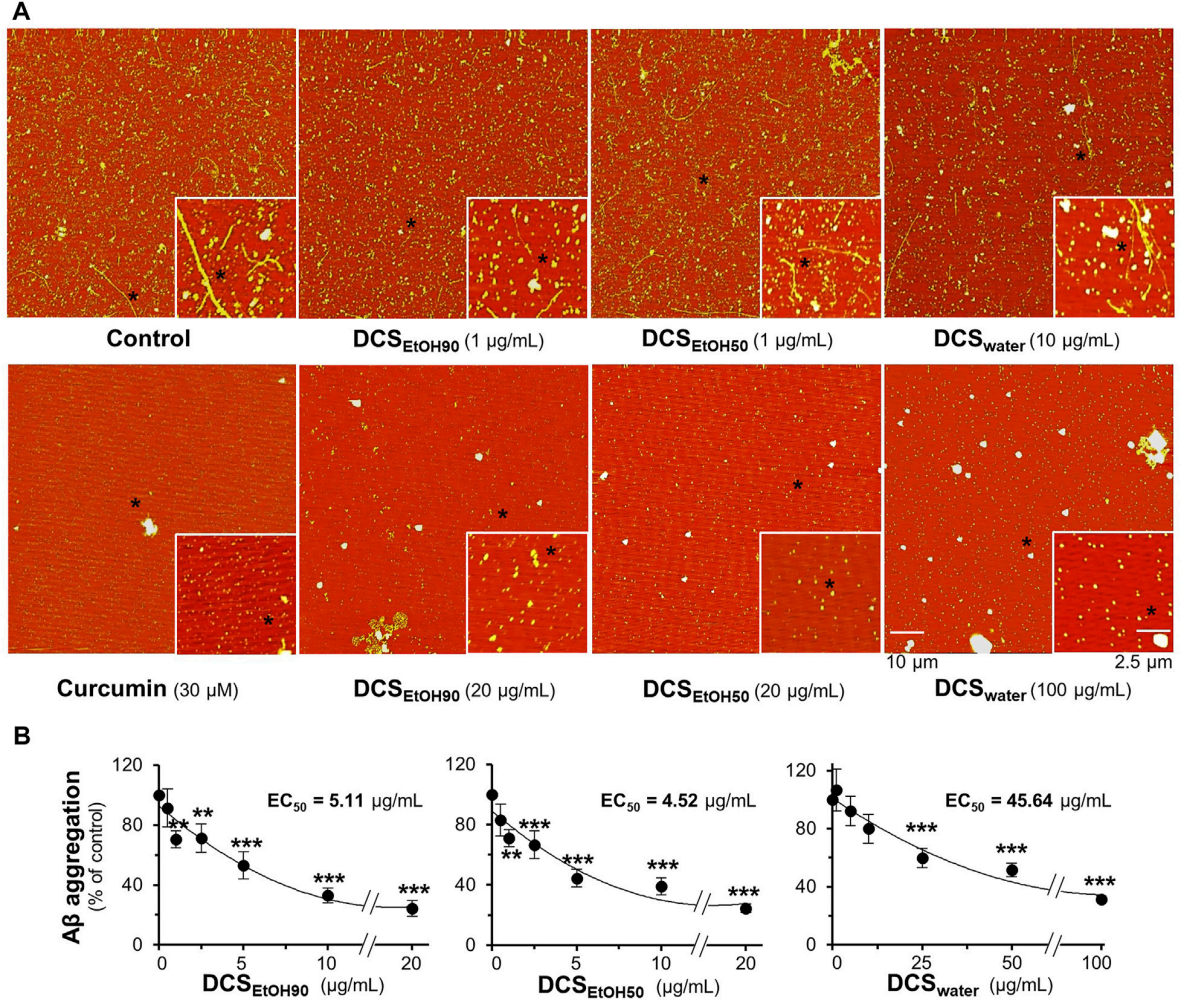
*D. cochinchinensis* stemwood extracts inhibit the formation of Aβ_1-42_ fibrils. Aβ_1-42_ monomers (10 μM) were incubated with or without DCS_EtOH90_ (0.1–20 μg/ml), DCS_EtOH50_ (0.1–20 μg/ml), or DCS_water_ (1–100 μg/ml) at 37°C for 6 days and then subjected to **(A)** AFM and **(B)** ThT fluorescent analysis. Data represent mean ± SEM for n = 4. **p < 0.01 and ***p < 0.001 as compared to the Aβ_1-42_-treated group.

To examine whether the extracts of *D. cochinchinensis* inhibited Aβ_1-42_ aggregation, Aβ_1-42_ monomer at 10 µM was co-aggregated with or without herbal extracts for 6 days, and then the Aβ aggregates were evaluated by AFM and ThT fluorescence assays. AFM images of Aβ_1-42_ co-aggregated with 1 μg/ml DCS_EtOH90_, 1 μg/ml DCS_EtOH50,_ or 10 μg/ml DCS_water_ showed fragmented Aβ_1-42_ fibrils (Figure 3A). Intact Aβ_1-42_ fibrils were no longer detected, while shorter Aβ_1-42_ fragments were sporadically distributed at 20 μg/ml DCS_EtOH90_, 20 μg/ml DCS_EtOH50,_ and 100 μg/ml DCS_water_ (Figure 3A). Curcumin was used as a positive control to reduce Aβ_1-42_ aggregation. In the ThT assay, treatment with DCS_EtOH90_, DCS_EtOH50,_ or DCS_water_ markedly prevented aggregation. The activity of the ethanol extract of *D. cochinchinensis* was more robust than that of the water extract (Figure 3B). DCS_EtOH90_ and DCS_EtOH50_ at 1–20 μg/ml concentrations, as well as DCS_water_ at 25–100 μg/ml concentration, significantly reduced Aβ_1-42_ aggregation in a dose-dependent manner (Figure 3B). The strength of inhibition exhibited by DCS_EtOH50_ with EC_50_ was approximately 4.52 μg/ml, DCS_EtOH90_ with EC_50_ was approximately 5.11 μg/ml, and DCS_water_ with EC_50_ was approximately 45.64 μg/ml.

**FIGURE 4:**
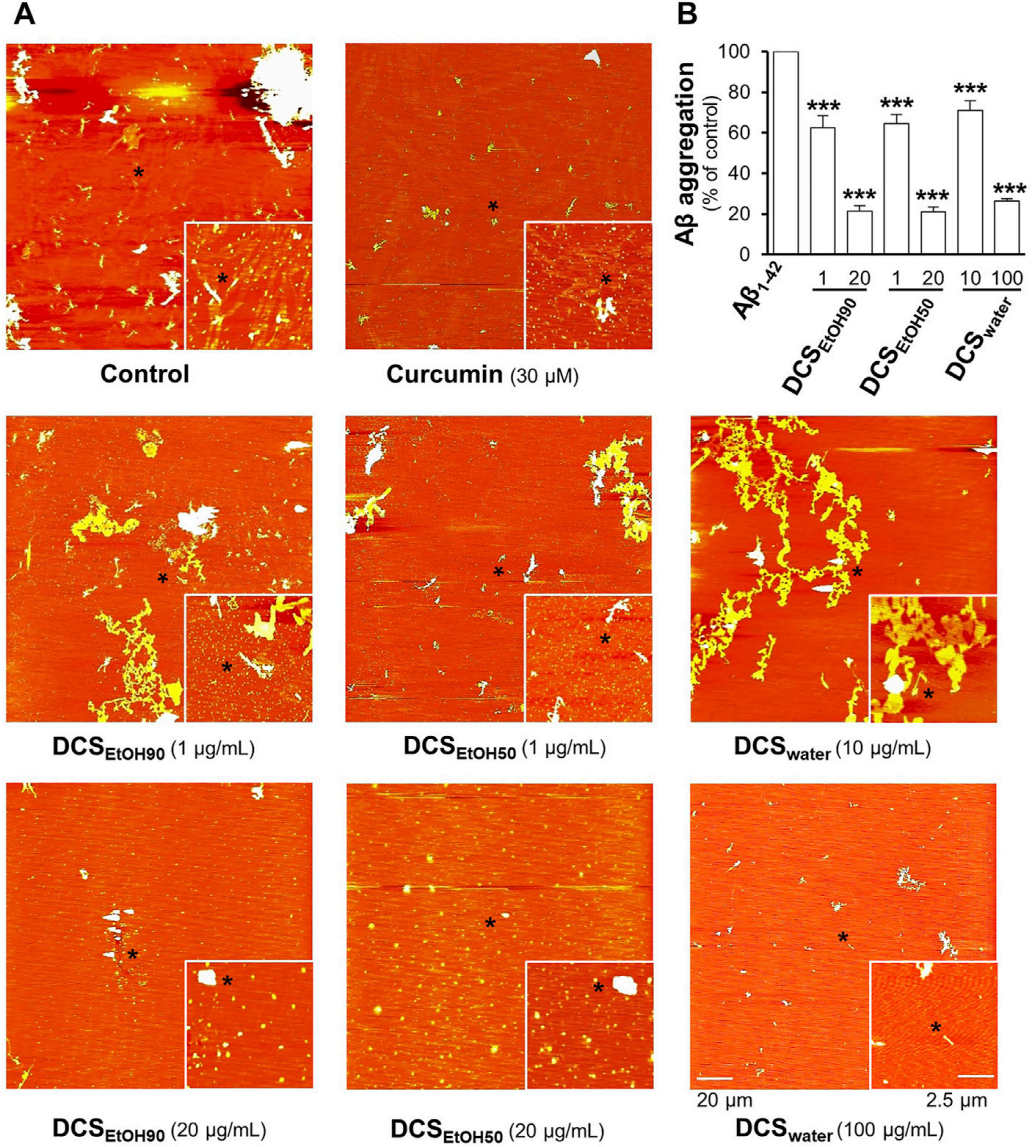
*D. cochinchinensis* stemwood extracts disassemble Aβ aggregation. Aβ_1-42_ fibrils (10 μM) aged for 6 days were incubated with or without DCS_EtOH90_ (1 and 20 μg/ml), DCS_EtOH50_ (1 and 20 μg/ml), or DCS_water_ (10 and 100 μg/ml) at 37°C for 5 days and then analyzed by **(A)** AFM and **(B)** ThT fluorescence assays. Values represent mean ± SEM for n = 4. ***p < 0.001 as compared to the Aβ_1-42_-treated group.

The extracts of *D. cochinchinensis* disrupted the formation of Aβ_1-42_ aggregates. In aged Aβ_1-42_ aggregates, the fibrils were dissociated by the applied herbal extracts in a dose-dependent manner, as revealed by AFM (Figure 4A). The dissociation properties were determined using the ThT fluorescent dye. The ethanol extracts (DCS_EtOH90_ and DCS_EtOH50_) showed better activity than the water extract (DCS_water_) (Figure 4B). This result suggests the recovery capability of *D. cochinchinensis* extracts in disassembling Aβ_1-42_ aggregates.

### Extracts of *D. cochinchinensis* stemwood promote neuronal survival and differentiation

**FIGURE 5:**
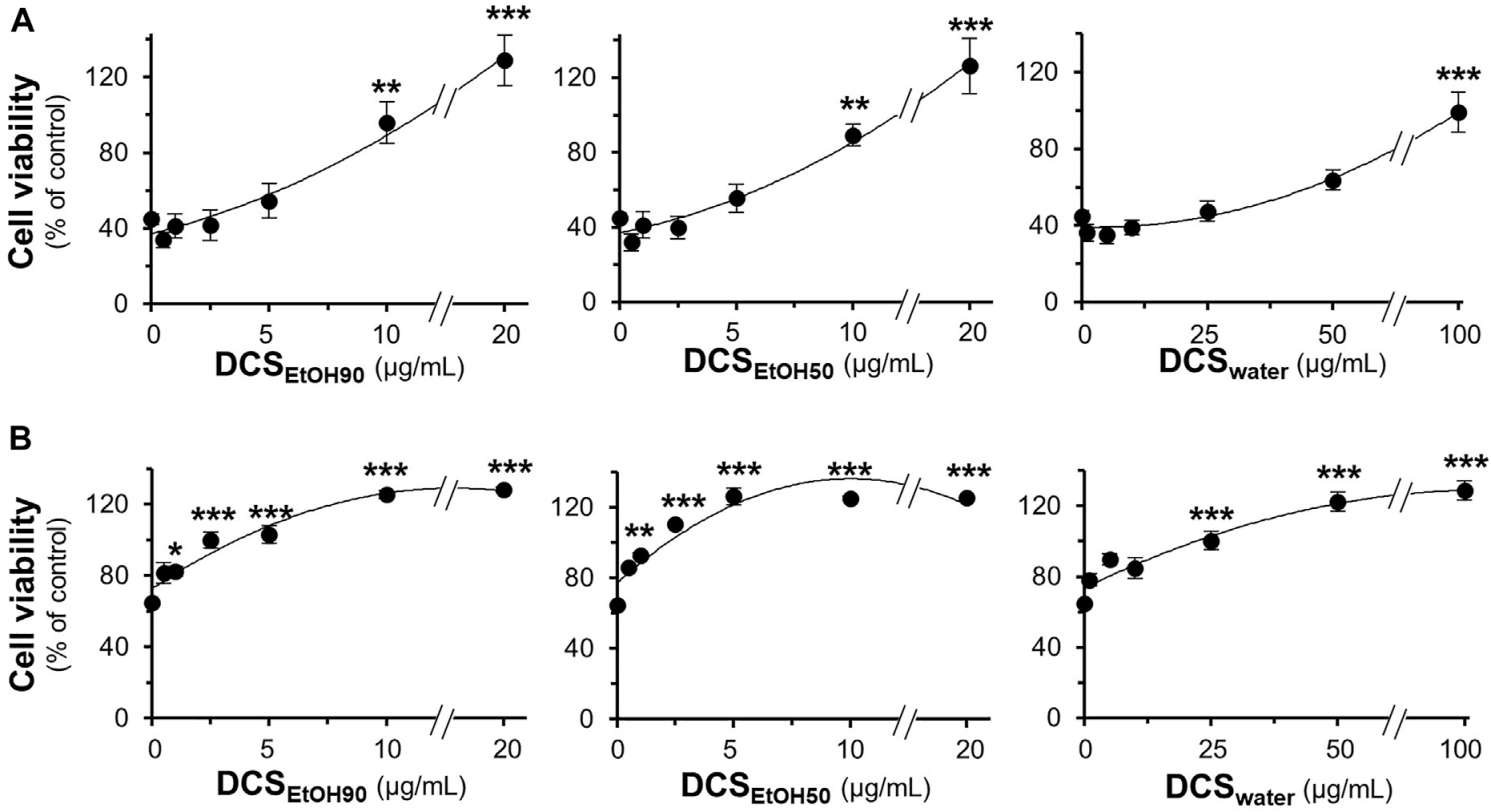
*D. cochinchinensis* stemwood extracts prevent Aβ-induced cell death. **(A)** Aβ_1-42_ monomers (10 μM) co-aggregated with or without DCS_EtOH90_ (0.1–20 μg/ml), DCS_EtOH50_ (0.1–20 μg/ml), or DCS_water_ (1–100 μg/ml) at 37°C for 6 days were applied to cultured PC12 cells for 24 h **(B)** PC12 cells were pre-treated with extracts for 24 h before exposure to Aβ_1-42_ fibrils (10 μM) aged 6 days for 24 h. Cell viability was measured using the MTT assay. Values represent mean ± SEM for n = 4. *p < 0.05, **p < 0.01, and ***p < 0.001 as compared to the untreated group.

The toxicities of DCS_EtOH90_, DCS_EtOH50,_ and DCS_water_ were determined in cultured PC12, BV2, and NG108-15 cells. After exposure of PC12 and NG108-15 cells to herbal extracts for 48 h, and BV2 cells for 24 h, it was observed that DCS_EtOH90_ and DCS_EtOH50_ significantly decreased cell viability at 25 μg/ml or above in PC12 cells, 50 μg/ml in NG108-15 cells, and 15 μg/ml in BV2 cells (Supplementary Figure S2). DCS_water_ did not decrease cell viability at concentrations up to 100 μg/ml. Thus, non-toxic doses of *D. cochinchinensis* extracts were used in the cell cultures. The extracts of *D. cochinchinensis* prevented cell death caused by Aβ_1-42_ fibrils. Two testing methods were used in this study. First, the Aβ_1-42_ monomers were co-incubated with *D. cochinchinensis* extracts for 6 days *in vitro* before the treatment of cultured cells for another 24 h. DCS_EtOH90_ and DCS_EtOH50_ showed robust restoration of cell death at 20 μg/ml, while DCS_water_ prevented cell death with less efficacy than the ethanol extracts (Figure 5A). Second, cultured cells were pre-treated with herbal extracts for 24 h before co-treatment with Aβ_1-42_ fibrils, at 6 days of aging, for another 24 h. DCS_EtOH90_ and DCS_EtOH50_ almost completely reduced Aβ_1-42_ fibril-induced cell toxicity at a concentration of 2.5 μg/ml (Figure 5B). DCS_water_ significantly prevented cell death at a concentration of 25 μg/ml. In both models, the extracts of *D. cochinchinensis* prevented Aβ_1-42_ fibril-induced cell death. Similar testing methods were performed in cultured BV2 and NG108-15 cells, and the protective role of *D. cochinchinensis* extracts was identified, similar to that in PC12 cells (Supplementary Figures S1B,C **)**.

**FIGURE 6:**
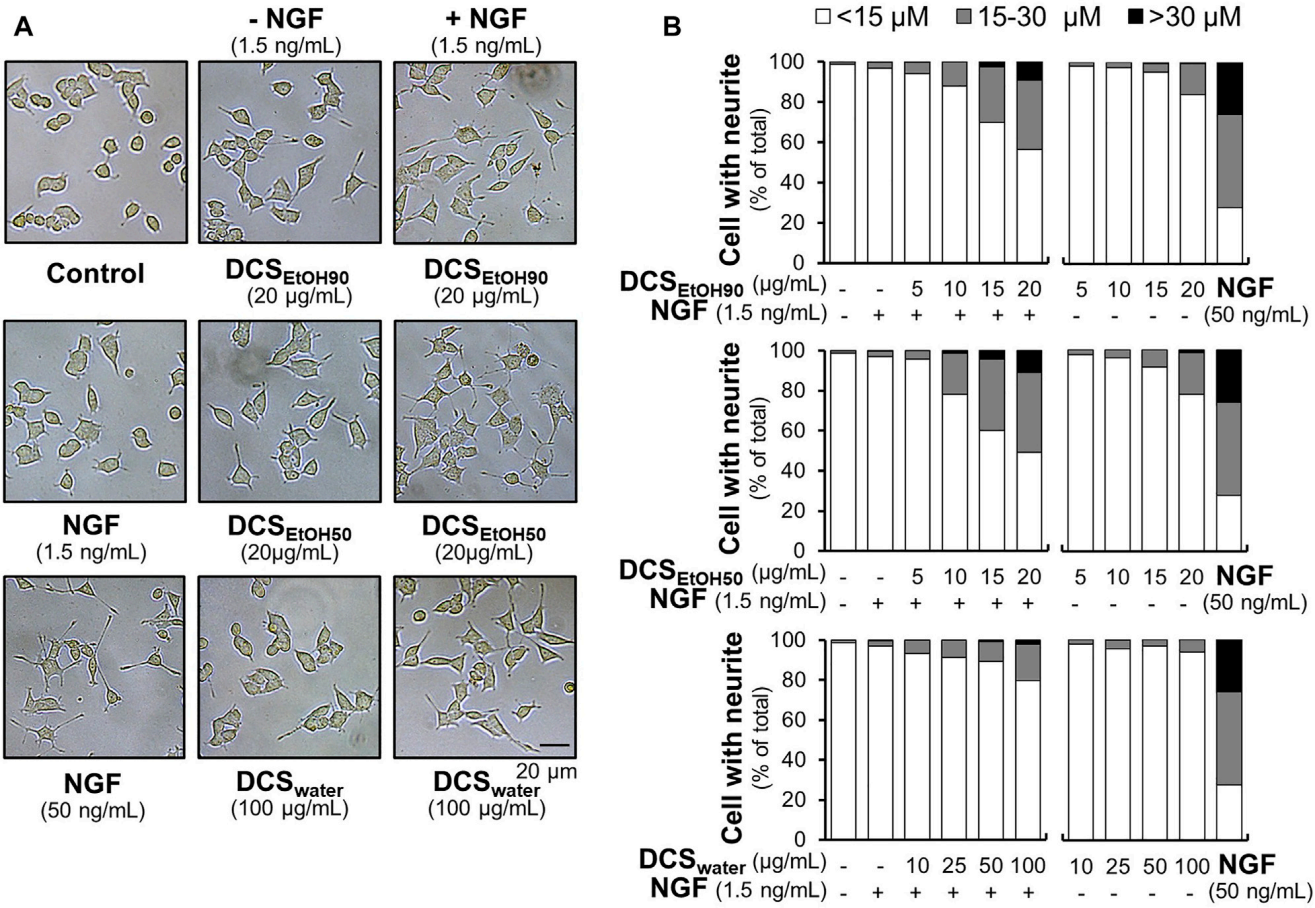
*D. cochinchinensis* stemwood extracts promote neurite outgrowth. **(A)** The morphology of PC12 cells was analyzed under a light microscope after 48 h treatment of DCS_EtOH90_, DCS_EtOH50_, or DCS_water_ with or without 1.5 ng/ml NGF. NGF at a concentration of 50 ng/ml was used as a positive control. **(B)** The length of the neurites was measured after 48 h of treatment with extracts in the presence or absence of 1.5 ng/ml NGF. The neurite length is presented as the percentage of cells with different neurite lengths in 100 counted cells, mean ± SEM for n = 4.

To study the ability of *D. cochinchinensis* extracts and to enhance neurite outgrowth in cultured PC12 cells, we first observed the morphological changes by measuring the neurite length, defined as <15 μm, 15–30 μm, and >30 µm. Under the conditions of serum starvation, cells were treated with *D. cochinchinensis* extracts in the presence or absence of a low dose of NGF at 1.5 ng/ml, which did not affect the neurite extension (Xu et al., 2019). NGF (50 ng/ml) was used as a positive control, inducing extensive neurite outgrowth (Figures 6A,B). Although the cultures treated with DCS_EtOH90_ and DCS_EtOH50_ exhibited neurite extensions, these neurites were shorter and less complex than those treated with 50 ng/ml NGF (Figures 6A,B). Co-treatment of the herbal extract with a low dose of NGF at 1.5 ng/ml robustly increased the extension of the neurites. The combination of the ethanol extracts of *D. cochinchinensis*, that is, DCS_EtOH90_ and DCS_EtOH50_, and a low dose of NGF was able to stimulate neurite outgrowth significantly, starting from 10 μg/ml of the extract. In addition, the ethanol extracts were more potent in promoting neurite growth than the water extract (DCS_water_) (Figures 6A,B).

**FIGURE 7:**
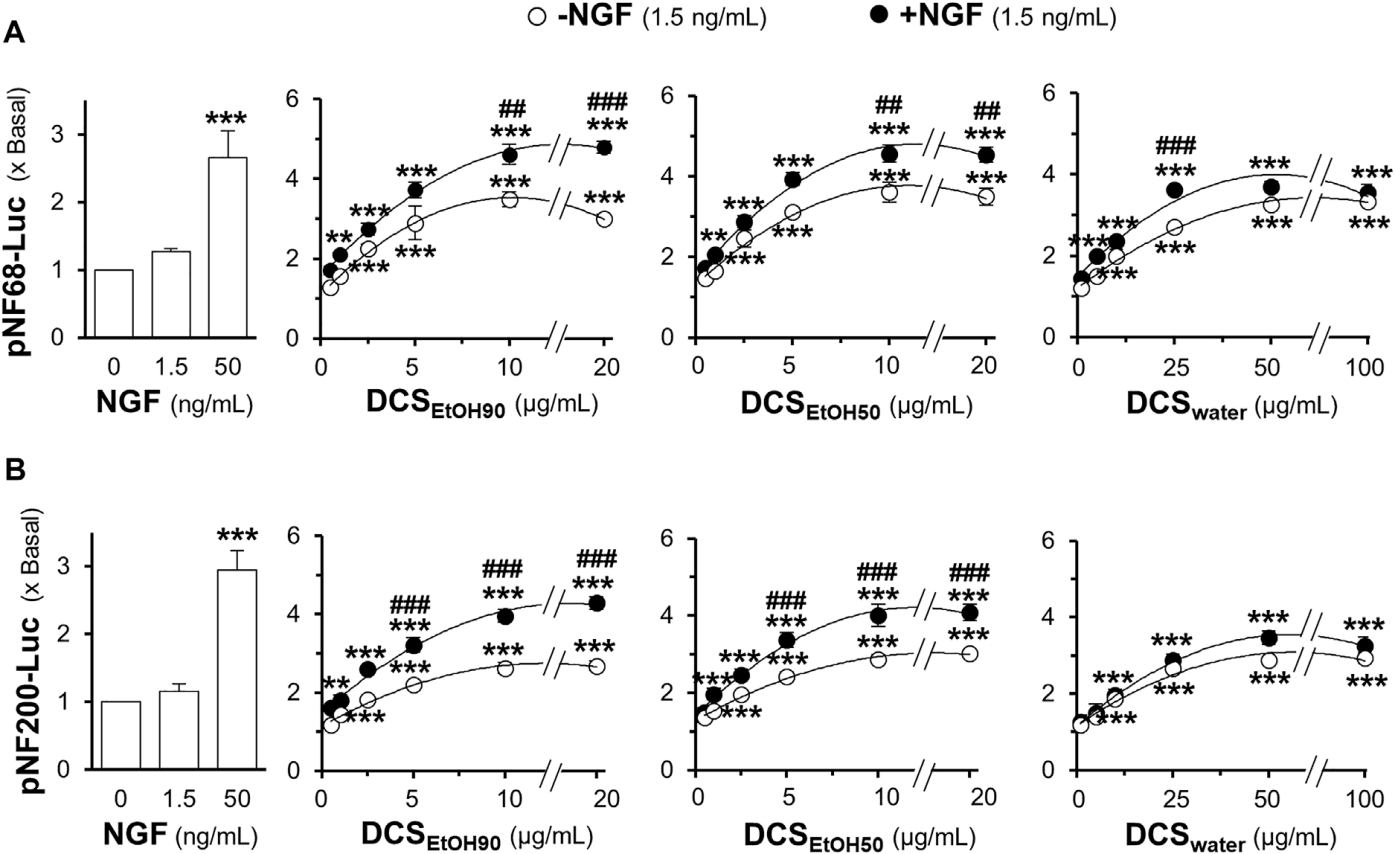
*D. cochinchinensis* stemwood extracts stimulate the transcriptional regulation of neurofilament promoters. Cultured PC12 cells transfected with the neurofilament promotor constructs pNF68-Luc and pNF200-Luc were treated with DCS_EtOH90_, DCS_EtOH50_, or DCS_water_ alone or in conjugation with 1.5 ng/ml NGF for 24 h. Cell lysates were collected for determining luciferase activity of **(A)** pNF68-Luc and **(B)** pNF200-Luc transfected cells. The luciferase activity is normalized to the amount of protein and represented as fold change, where mean ± SEM for n = 4. **p < 0.01 and ***p < 0.001 as compared to untreated cells. ##*p* < 0.01 and ###*p* < 0.001 as compared to cells treated with the extracts in the absence of 1.5 ng/ml NGF.

Neurofilament expression is closely associated with neurite outgrowth. Here, a luciferase reporter gene was tagged downstream of the neurofilament promoters, pNF68-Luc and pNF200-Luc. The plasmid was transfected into cultured PC12 cells. NGF (1.5 ng/ml) did not show any significant effect on the promoter activities of pNF68-Luc and pNF200-Luc (Figures 7A,B). However, NGF, at a concentration of 50 ng/ml, showed robust induction. Without a low dose of NGF, the extracts of *D. cochinchinensis* induced luciferase activity in a dose-dependent manner. Maximal induction was achieved with 10 μg/ml of ethanol extract and 100 μg/ml of water extract. Co-treatment with a low dose of NGF markedly increased the luciferase activity in both DCS_EtOH90_ and DCS_EtOH50_ (Figures 7A,B). The combination of DCS_water_ and low dose of NGF-inducing luciferase activity was weak.

**FIGURE 8:**
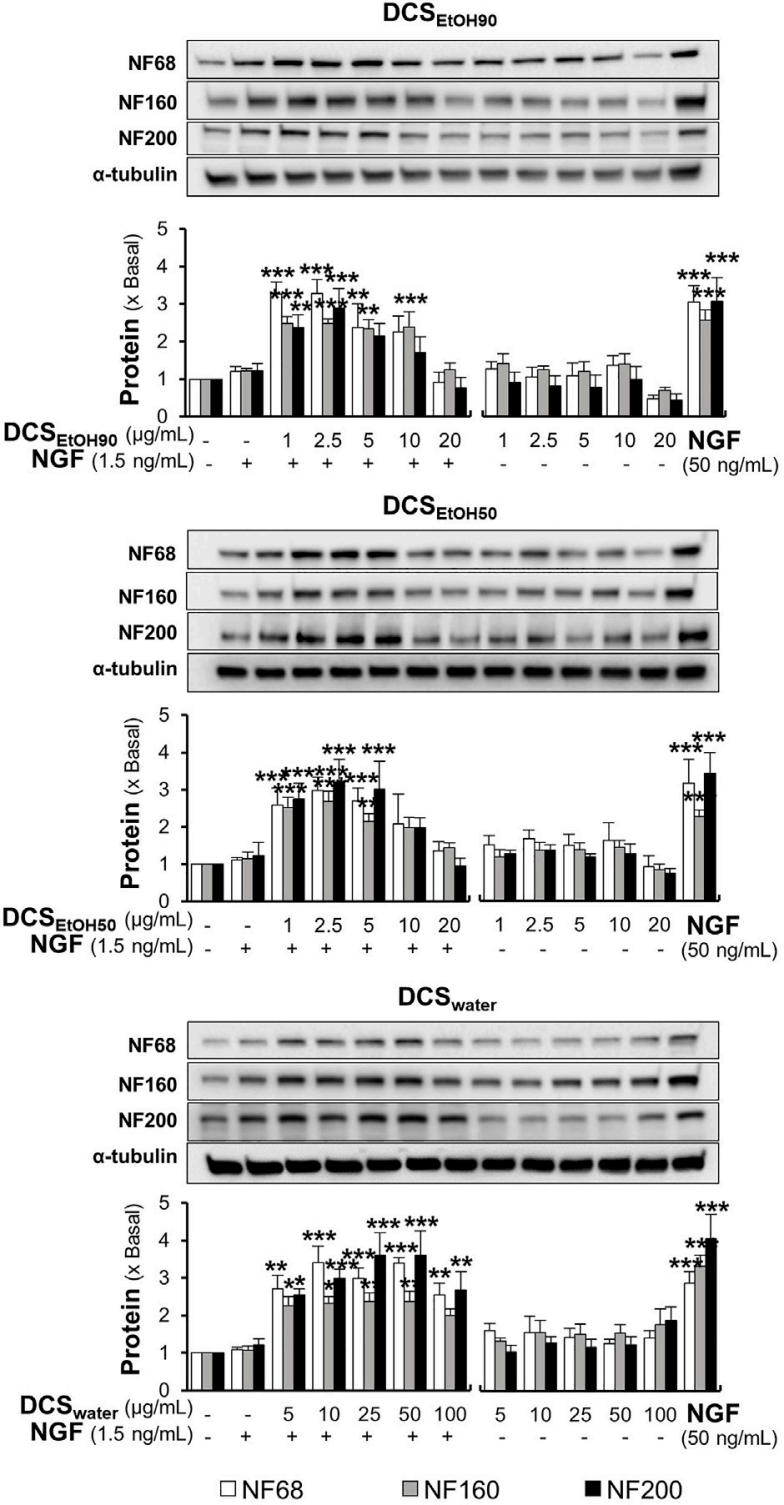
*D. cochinchinensis* stemwood extracts and a low dose of NGF synergistically induce neurofilament protein expression. Cells were exposed to DCS_EtOH90_, DCS_EtOH50_, or DCS_water_ with or without 1.5 ng/ml NGF for 48 h. Cells were lysed and the amount of neurofilament protein, i.e., NF68, NF160, and NF200, was quantified by Western blotting. The data are demonstrated as fold change of mean ± SEM, n = 4. *p < 0.05, **p < 0.01, and ***p < 0.001 as compared to the untreated cells.

In parallel, the protein expression levels of neurofilaments (NF) NF68, NF160, and NF200 were determined by Western blotting. As expected, a low dose of NGF (1.5 ng/ml) did not show any significant change in the protein expression of NF68 (approximately 68 kDa), NF160 (approximately 160 kDa), and NF200 (approximately 200 kDa) (Figure 8). In comparison, a higher concentration of NGF at 50 ng/ml induced protein expression three-to four-folds. Moreover, the synergy of 1.5 ng/ml NGF and *D. cochinchinensis* extracts increased the protein expression by three-to four-folds. Here, DCS_EtOH90_ and DCS_EtOH50_ at 1 μg/ml and DCS_water_ at 5 μg/ml, which were the lowest concentrations combined with a low dose of NGF, significantly induced the protein expression (Figure 8).

### Major phytochemicals of *D. cochinchinensis* stemwood ameliorating Aβ_1-42_ fibrils and cell survival

**FIGURE 9:**
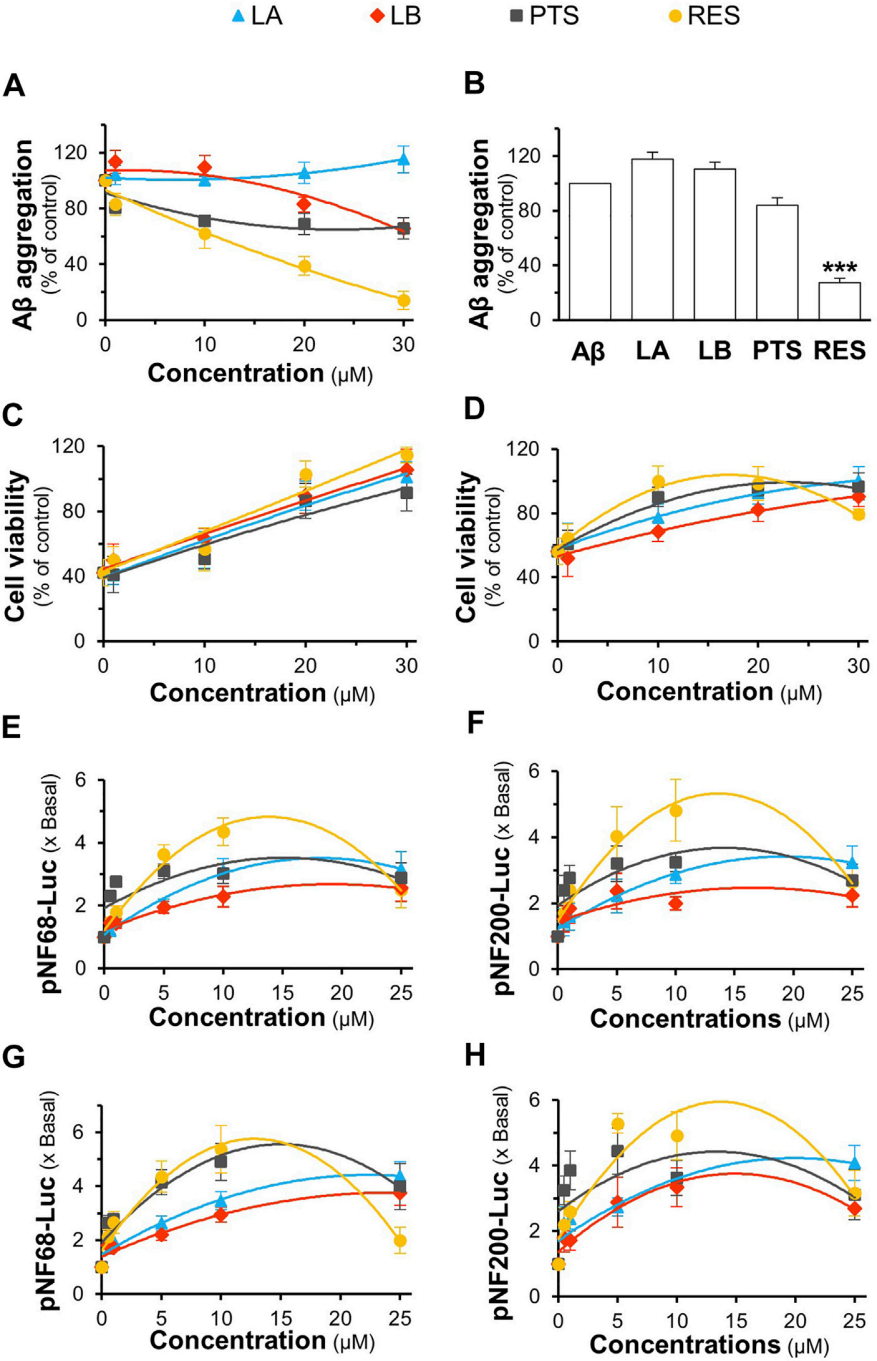
Role of *D. cochinchinensis* stemwood extract phytochemicals in inhibiting Aβ aggregation and promoting cell viability and differentiation in PC12 cells. **(A)** Blockage of Aβ_1-42_ fibril formation: Aβ_1-42_ monomers (10 μM) were aggregated or co-aggregated with the different concentrations (1–30 μM) of loureirin A (LA), loureirin B (LB), pterostilbene (PTS), or resveratrol (RES) at 37°C for 6 days **(B)** Disruption of Aβ aggregation: The aged Aβ fibrils (10 μM at 6-days) were incubated with or without these four compounds (30 μM) at 37°C for 5 days, then subjected to the ThT fluorescent assay. **(C)** The Aβ_1-42_ monomers aggregates (10 μM) with or without the phytochemicals at 1–30 μM for 6 days were applied to PC12 cells for 24 h **(D)** PC12 cells were pre-treated with these phytochemicals for 24 h and exposed to Aβ_1-42_ fibrils (10 μM; aged for 6 days) for 24 h. The cell viability was determined by the MTT assay. **(E,F)** pNF68-Luc or pNF200-Luc transfected PC12 cells were treated with concentrations of 0.5, 1, 5, 10, and 25 μM of the compounds for 24 h **(G,H)** The pNF68-Luc- or pNF200-Luc-transfected PC12 cells were co-treated with phytochemicals and 1.5 ng/ml NGF for 24 h. Luciferase activity of pNF68-Luc and pNF200-Luc is represented as fold change (x Basal) to untreated cells. All values are in mean ± SEM for n = 4.

Loureirin A, loureirin B, pterostilbene, and resveratrol are the major constituents of *D. cochinchinensis* extracts, accounting for the functionality of herbal extracts, as reported here. We first examined their safe concentrations in cell culture using the MTT assay. Loureirin A, loureirin B, pterostilbene, and resveratrol at concentrations less than or equal to 30 μM were chosen for cell culture experiments, where cell damage was not identified after 48 h of treatment (Supplementary Figure S3). Consequently, loureirin A, loureirin B, pterostilbene, and resveratrol, at concentrations of 1–30 μM, were co-incubated with 10 μM Aβ_1-42_ monomers at 37°C for 6 days. Resveratrol significantly inhibited Aβ_1-42_ aggregation in a dose-dependent manner. The complete suppression of Aβ_1-42_ aggregation was observed after treatment with 30 μM resveratrol (Figure 9A). Loureirin B and pterostilbene showed weak activities, whereas loureirin A had no inhibitory effect on Aβ_1-42_ aggregation (Figure 9A). At a similar potency, 30 μM resveratrol was the only phytochemical that was able to resolve the aggregation of Aβ_1-42_ (Figure 9B).

To determine the protective effect of loureirin A, loureirin B, pterostilbene, and resveratrol, Aβ_1-42_ monomers were co-incubated with the compounds for 6 days before being applied to cultured PC12 cells for 24 h. The four chemicals prevented cell death in a dose-dependent manner, with resveratrol showing the best activity (Figure 9C). In addition, cultures pretreated with loureirin A, loureirin B, pterostilbene, and resveratrol before treatment with Aβ_1-42_ fibrils prevented Aβ_1-42_ fibril-induced cytotoxicity in a dose-dependent manner (Figure 9D). Resveratrol showed the best cell protection in both scenarios.

**FIGURE 10:**
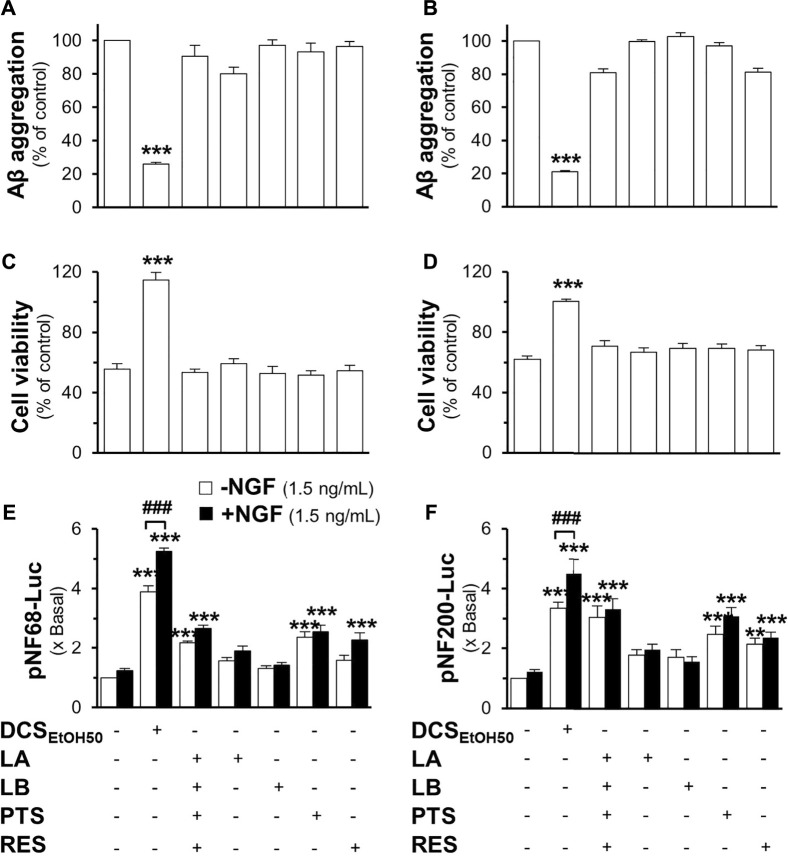
The mixture of major compounds does not account for the activity of the *D. cochinchinensis* stemwood extract. The activity of loureirin A (LA; 0.8 μM), loureirin B (LB; 0.7 μM), pterostilbene (PTS; 1.1 μM), or resveratrol (RES; 1.2 μM), with 20 μg/ml DCS_EtOH50_ as well as DCS_EtOH50_ (20 μg/ml), was analyzed. **(A)** Inhibition of Aβ_1-42_ fibrilization: Aβ_1-42_ monomers (10 μM) were incubated with these compounds and DCS_EtOH50_ for 6 days **(B)** The reversal of Aβ aggregates: Aβ fibrils (10 μM; aged 6 days) were treated with the compounds or herbal extracts for 5 days, and Aβ aggregation was measured using ThT fluorescence. **(C)** Cells were treated with 6-days co-aggregated Aβ_1-42_ monomers (10 μM) and the compounds or extracts for 24 h. **(D)** Cells were pre-treated with the compounds or extracts for 24 h before exposure to Aβ fibrils (10 μM). Cell viability was measured by using the MTT assay. **(E)** Cells were transfected with pNF68-Luc or **(F)** pNF200-Luc before treatment with the compounds or the extract in the presence or absence of 1.5 ng/ml NGF for 24 h, and then subjected to the luciferase analysis. Data are presented as mean ± SEM for n ≥ 4. **p < 0.01 and ***p < 0.001 as compared to the untreated groups. ##*p* < 0.01 and ###*p* < 0.001 as compared to cells treated with the extract without 1.5 ng/ml NGF.

Loureirin A, loureirin B, pterostilbene, and resveratrol were applied to pNF68-Luc and pNF200-Luc transfected PC12 cells, with or without a low dose of NGF. Without the combination of 1.5 ng/ml NGF, these chemicals induced promoter activity in a dose-dependent manner, where resveratrol was the best inducer (Figures 9E,F). In another approach, promoter DNA-transfected PC12 cells were treated with low doses of NGF and the *D. cochinchinensis* chemicals loureirin A, loureirin B, pterostilbene, and resveratrol. Promoter induction by co-treatment was robust with over a five-fold luciferase activity (Figures 9G,H). These *D. cochinchinensis* chemicals accounted for the functionality of the herbal extracts; however, the amounts of loureirin A, loureirin B, pterostilbene, and resveratrol in the *D. cochinchinensis* extracts could not account for the identified activities (Supplementary Table S1), which were much lower than their functional range in the assays. The four chemicals contained in 20 μg/ml of DCS_EtOH50_ were mixed and applied to different assays to mimic the herbal extract. In all scenarios, the synergy of the four chemicals did not mimic the activities of DCS_EtOH50_ (Figures 10A–F). The individual compounds resveratrol and pterostilbene showed minor induction of pNF68-Luc and pNF200-Luc, showing lower activity than 20 μg/ml DCS_EtOH50_ (Figures 10E,F). In addition, synergy with a low NGF dose did not occur. These results suggest a unique distinction of the herbal mixture.

## Discussion

The incidence of AD is increasing, although its pathogenesis remains unclear. One of the proposed AD hypotheses is known as “amyloid hypothesis”. The aggregation of Aβ peptides and their deposition in the brain have been proposed as causes of medical problems (Murphy and LeVine, 2010; Chen et al., 2017). The blockade of Aβ aggregation is one of the several therapeutic strategies for AD treatment. Natural products are considered an important source of bioactive compounds that are believed to have minimal side effects (Kumar and Nisha, 2014; Pagano et al., 2020). The stemwood of *D. cochinchinensis* has been used as an herbal medicine in Thailand for years; however, pharmacological studies of this herb in terms of its neuroprotective and neurotrophic abilities are very limited. The extracts of *D. cochinchinensis* inhibited and disassembled Aβ aggregation. In addition, our results showed a dose-dependent correlation between the *D. cochinchinensis* extracts in reducing Aβ fibril formation and the loss of cell viability. These findings indicate that the *D. cochinchinensis* extracts were able to inhibit Aβ fibril formation and clear Aβ aggregation, as well as prevent Aβ fibril-induced cell death. The neuroprotective role of the *D. cochinchinensis* extracts, however, was not associated with the pro-survival signaling of Akt, CREB, and Erk1/2 (Supplementary Figure S4). The efficacy of the ethanol extracts in inhibiting Aβ fibril formation, disassembling Aβ aggregation, and defending against cell death was approximately ten-fold higher than that of the water extract, which may be accounted for by higher amounts of active compounds, such as resveratrol, in the ethanol extracts. Although resveratrol has been tested for pharmacological activities, its concentration is not sufficient to account for all the properties of the *D. cochinchinensis* extracts. In addition to resveratrol, the chemical compositions of the *D. cochinchinensis* extracts have been reported, for example, loureirin A, loureirin B, loureirin C, loureirin D, 7,4′-dihydroxyflavone, homoisoflavones, retrodihydrochalcones, stilbenoids, and flavonoid derivatives (Meksuriyen and Cordell, 1988; Ichikawa et al., 1997; Likhitwitayawuid et al., 2002). However, the role of these phytochemicals in AD treatment has not been fully elucidated.

Aggregated Aβ triggers the production of pro-inflammatory cytokines from microglia and astrocytes, leading to neuroinflammation and brain damage (Jin et al., 2008; Garwood et al., 2011). In line with this notion, rutin, a flavonoid, has been reported to inhibit Aβ fibrillization and reduce proinflammatory cytokines such as TNF-α and IL-1β produced by the microglia (Wang et al., 2012). Therefore, the extracts of *D. cochinchinensis* possibly provide neuroprotection by suppressing inflammatory responses in the brain. Among the four identified compounds in the *D. cochinchinensis* extracts represented in the HPLC chromatograms, we found that resveratrol was the most abundant compound in both the ethanol and water extracts, followed by pterostilbene, loureirin A, and loureirin B. Resveratrol and pterostilbene are prospective candidates as anti-aging agents that modulate aging hallmarks, such as oxidative stress and inflammation (Li et al., 2018). The pharmacological activities of resveratrol have been reported in AD models, for example, increasing the clearance of Aβ peptides and reducing Aβ aggregation as well as enhancing neurite outgrowth and synaptogenesis (Jia et al., 2017; Tang et al., 2017). Additionally, pterostilbene exhibited neuroprotective properties by restoring Aβ-induced cognitive dysfunction in a mouse model (Xu et al., 2021). The activities of loureirins A and B in neurodegenerative diseases have not been reported; however, these chemicals have shown anti-inflammatory effects in other disease models (Hu W. H. et al., 2020; Sun et al., 2020). Moreover, loureirin B promotes axon regeneration and neuron polarization in rats with spinal cord injury (Wang et al., 2019).

The current results support the theory that the mature forms of Aβ fibrils induce neuronal cell death. Low concentrations of Aβ_1-42_ (0.1 and 1 µM) were toxic; however, these concentrations did not show aggregation. It is possible that small amounts of Aβ peptides inhibited the formation of fibrils or halted fibril identification (Legleiter and Kowalewski, 2004). Treatment with *D. cochinchinensis* ethanol and water extracts at 2.5 and 25 μg/ml, respectively, completely prevented Aβ fibril-induced cell death. Resveratrol, pterostilbene, loureirin B, and loureirin A were able to perform similarly to the *D. cochinchinensis* extracts, at least partly in the range of 10–30 μM. However, these effective concentrations were much higher than those of the herbal extracts (μg/mg), as described here. Other chemicals or synergies of these chemicals with the herbal extract are hypothesized to have functional roles. Interestingly, loureirin A did not inhibit Aβ aggregation; however, it prevented Aβ fibril-induced cell death. Several natural compounds and herbal medicines have been reported to prevent Aβ-induced cell death through different signaling pathways, including anti-apoptosis, anti-autophagy, anti-oxidative damage, and anti-inflammation pathways (Chen et al., 2020).

Memory impairment and cognitive decline in AD have been reported to be associated with the loss of cholinergic neurons and/or cholinergic dysfunction. The amyloid hypothesis of AD proposes that neuronal dysfunction might act through the decreased neurite outgrowth of neurons (Petratos et al., 2008). In contrast, several lines of evidence suggest that the synergy of Aβ with laminin and fibronectin promotes neuronal differentiation (Koo, et al., 1993; Saad et al., 2015). Here, the aggregated Aβ did not affect the morphology and neurite change in PC12 cells, and there was no effect of the Aβ and NGF combination in enhancing differentiation (Supplementary Figure S5). NGF is the major tropic factor that supports cholinergic neuronal integrity and function, as well as modulates the neurogenesis and neurite outgrowth during neuronal development (Hefti and Weiner, 1986; Mitra et al., 2019). A reduction in NGF is found in the brains of patients with neurodegenerative diseases. However, it is a protein that is unable to pass the BBB (Lärkfors et al., 1987; Bartus, 2000; Tuszynski et al., 2005; Budni et al., 2015). Here, the synergy of the *D. cochinchinensis* extract with a low dose of NGF is shown to stimulate neurite outgrowth and the expression of neurofilaments. Neurofilaments maintain neuronal integrity and differentiation (Carden et al., 1987; Khalil et al., 2018). Thus, *D. cochinchinensis* extracts may be used to treat neurodegenerative diseases in those with insufficient amounts of NGF. Previous studies have reported that resveratrol enhances neurite outgrowth (Dasgupta and Milbrandt, 2007; Tang et al., 2017). Resveratrol is a small molecule that can pass the BBB. However, only a small amount of resveratrol accumulates in the brain after oral intake, which is insufficient to reach a therapeutic dosage (Poulose et al., 2015). In addition, patients with AD receiving resveratrol by oral administration did not show promising results due to their low bioavailability (Arbo et al., 2020). In contrast, pterostilbene has a similar structure to resveratrol but with good liposolubility, suggesting possible penetration into the BBB (Poulose et al., 2015; Liu et al., 2020). Consistently, the current results revealed that resveratrol and pterostilbene were able to induce the protein expression of neurofilaments and play a synergistic role with NGF. This notion validates the extensive work on this Thai herbal medicine, particularly the possibility of these phytochemicals penetrating the BBB.

## Data Availability

The original contributions presented in the study are included in the article/Supplementary Material; further inquiries can be directed to the corresponding author.

## Abbreviations

Aβ, Amyloid-β; AD, Alzheimer’s disease; AFM, atomic force microscope; Akt, protein kinase B; BBB, blood-brain barrier; CREB, cAMP response element-binding protein; DCS, *D. cochinchinensis* stemwood; Erk1/2, extracellular signal-regulated kinase 1/2; FBS, fetal bovine serum; HPLC, high-performance liquid chromatography; NGF, nerve growth factor; NF, neurofilament.
