# Supplementary material for: Dracaena cochinchinensis stemwood extracts inhibit amyloid-β fibril formation and promote neuronal cell differentiation
Source: Front Pharmacol. 2022 Sep 6;13:943638. doi: 10.3389/fphar.2022.943638 (PMC9486383; doi:10.3389/fphar.2022.943638)
Supplement: Supplementary file 1 [file DataSheet1.pdf]

Supplementary Material

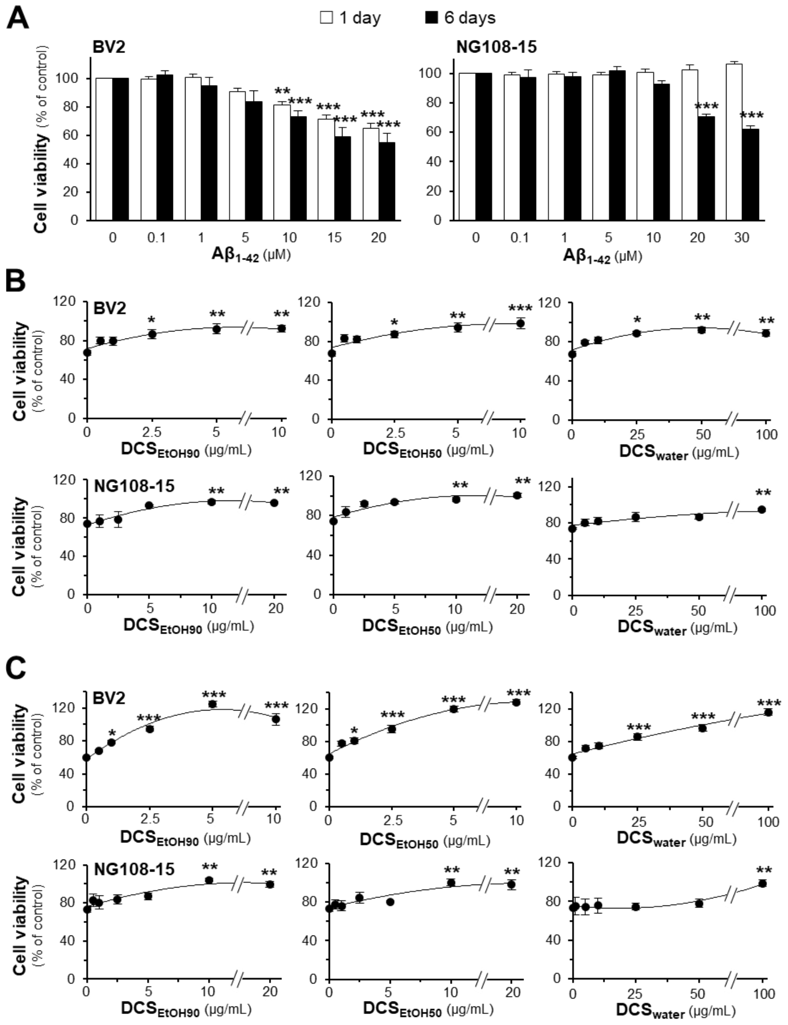

Supplementary Figure 1. ***D. cochinchinensis*** **stemwood extracts prevent Aβ-induced cell death.** **(A)** $A\beta_{1\text{-}42}$ (0.1-30 μM) aggregates at 1 and 6 d were applied to BV2 and NG108-15 cells for 24 h. **(B)** $A\beta_{1\text{-}42}$ monomers (15 μM for BV2 cells and 20 μM for NG108-15 cells) co-aggregated with or without DCS_{EtOH90} (0.5-20 μg/mL), DCS_{EtOH50} (0.5-20 μg/mL) or DCS_{water} (5-100 μg/mL) at 37 °C for 6 d were applied to cultured cells for 24 h. **(C)** BV2 and NG108-15 cells were pre-treated with extracts for 4 and 24 h, respectively, before exposure to $A\beta_{1\text{-}42}$, i.e., 15 and 20 μM, respectively, aged 6 d for 24 h. Cell viability was measured using the MTT assay. Values represent mean ± SEM for $n = 4$. * $p < 0.05$, ** $p < 0.01$, and *** $p < 0.001$ as compared to untreated group.

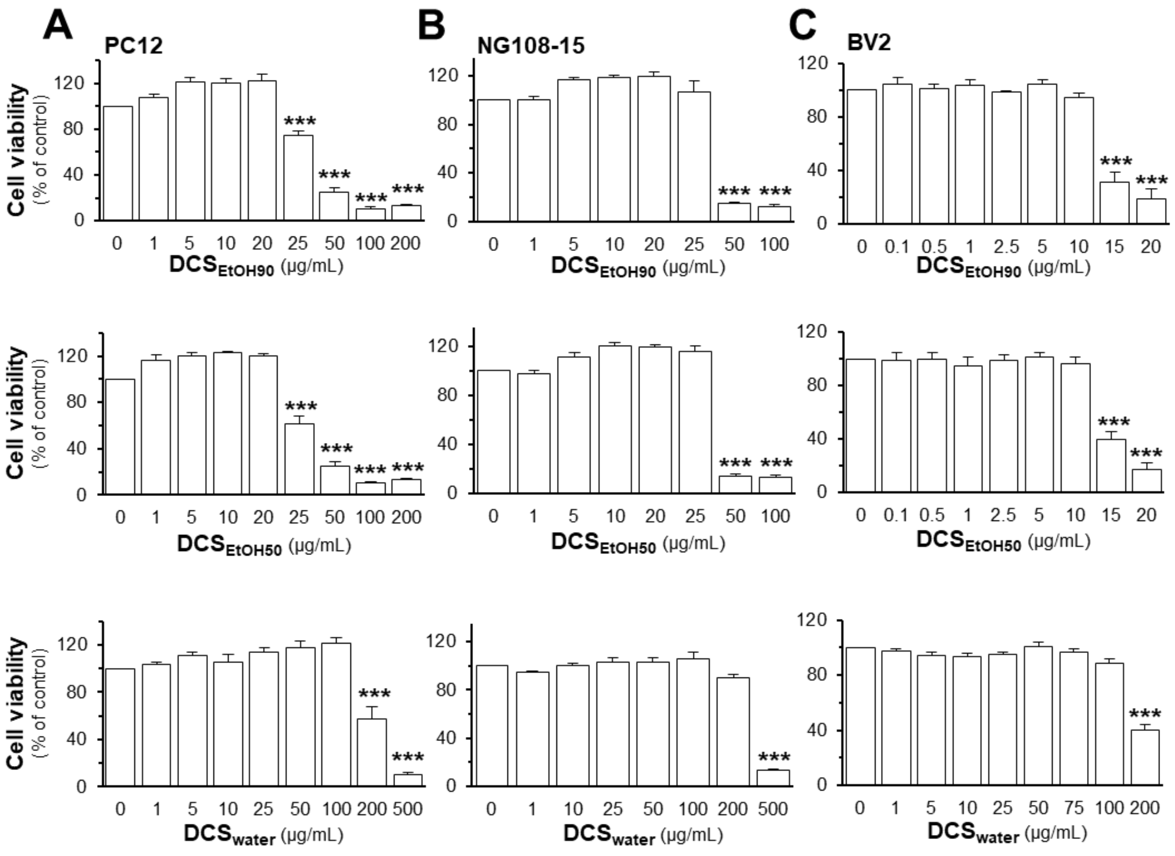

Supplementary Figure 2. Cytotoxicity of *D. cochinchinensis* stemwood extracts. (A) Cultured PC12 and **(B)** NG108-15 cells were treated with different concentrations of DCS_{EtOH90}, DCS_{EtOH50}, and DCS_{water} for 48 h. **(C)** BV2 cells were treated with DCS_{EtOH90}, DCS_{EtOH50}, and DCS_{water} for 24 h. MTT assay was used as an indicator of cell viability. Data are represented as mean ± SEM for $n = 4$. *** $p < 0.001$ as compared to untreated cells.

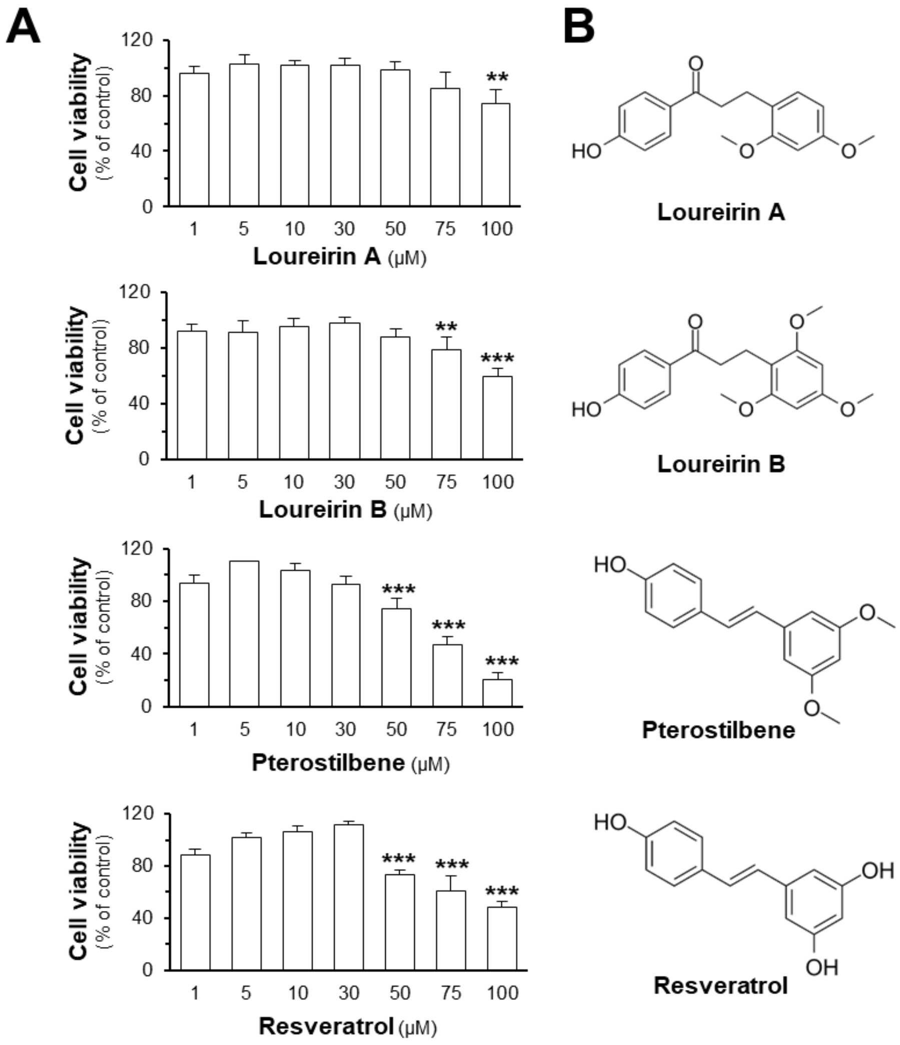

Supplementary Figure 3. Cytotoxicity of *D. cochinchinensis* stemwood extract phytochemicals (A) PC12 cells were treated with various concentrations of loureirin A, loureirin B, pterostilbene, or resveratrol for 48 h, and analyzed by the MTT assay. Values represent mean ± SEM for $n = 4$. ** $p < 0.01$, and *** $p < 0.001$ as compared to untreated cells. **(B)** Chemical structures of loureirin A, loureirin B, pterostilbene and resveratrol.

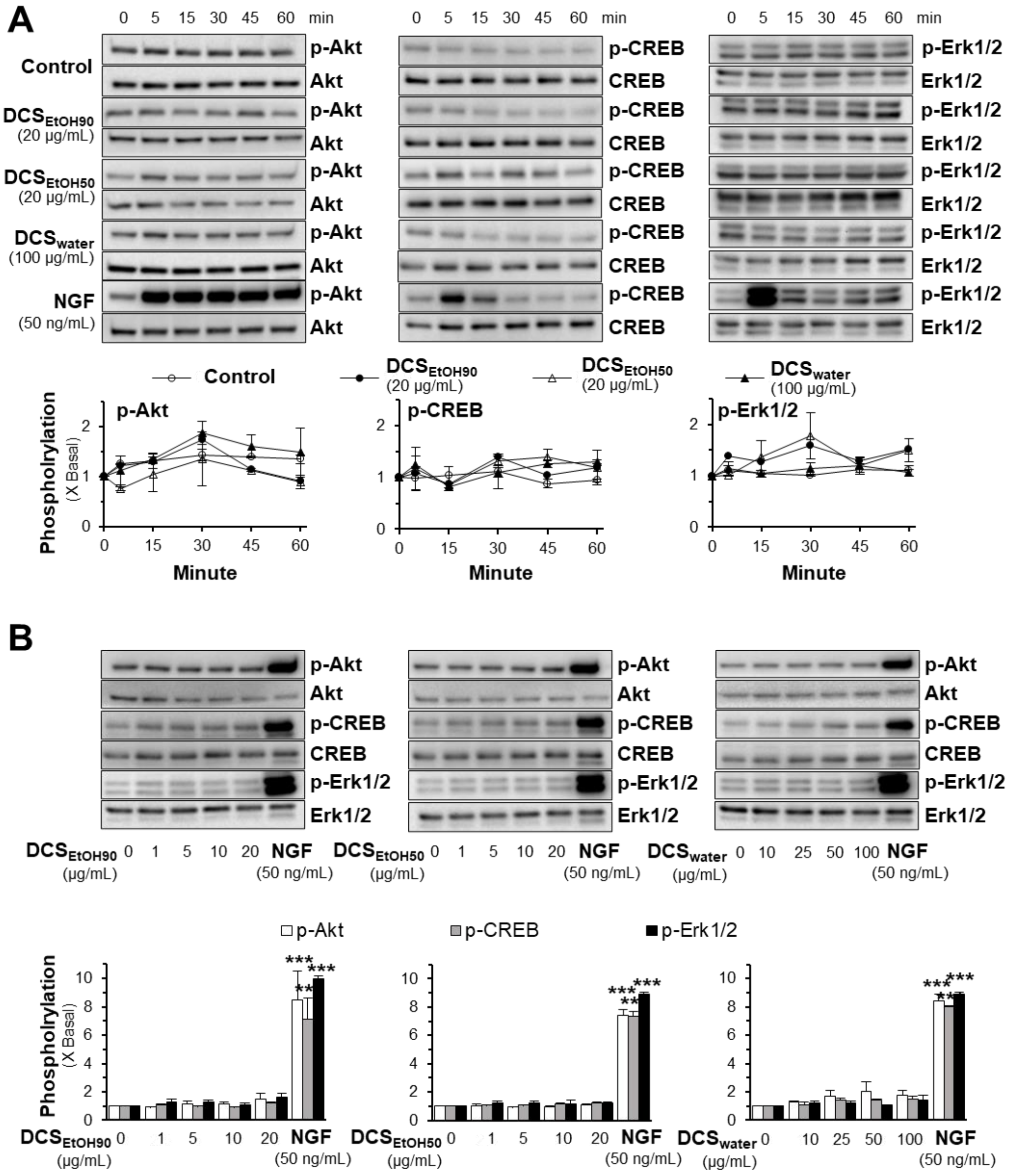

Supplementary Figure 4. Phosphorylation of pro-survival Akt, CREB, and Erk1/2 signaling pathway under the herbal treatment. The PC 12 cultures were serum-starved for 24 h. **(A)** Cells were treated with DCS_{EtOH90} (20 µg/mL), DCS_{EtOH50} (20 µg/mL) or DCS_{water} (100 µg/mL) at various time points. **(B)** Cells were treated with various concentrations of DCS_{EtOH90}, DCS_{EtOH50} or DCS_{water} for 5 min. NGF at a concentration of 50 ng/mL was used as a positive control. Cells were immediately harvested for western blotting analysis of total and phosphorylated Akt (approximately 60 kDa), CREB (approximately 40 kDa), and Erk1/2 (approximately 42/44 kDa). Values are mean ± SEM for $n = 4$. *** $p < 0.001$ as compared to untreated cells.

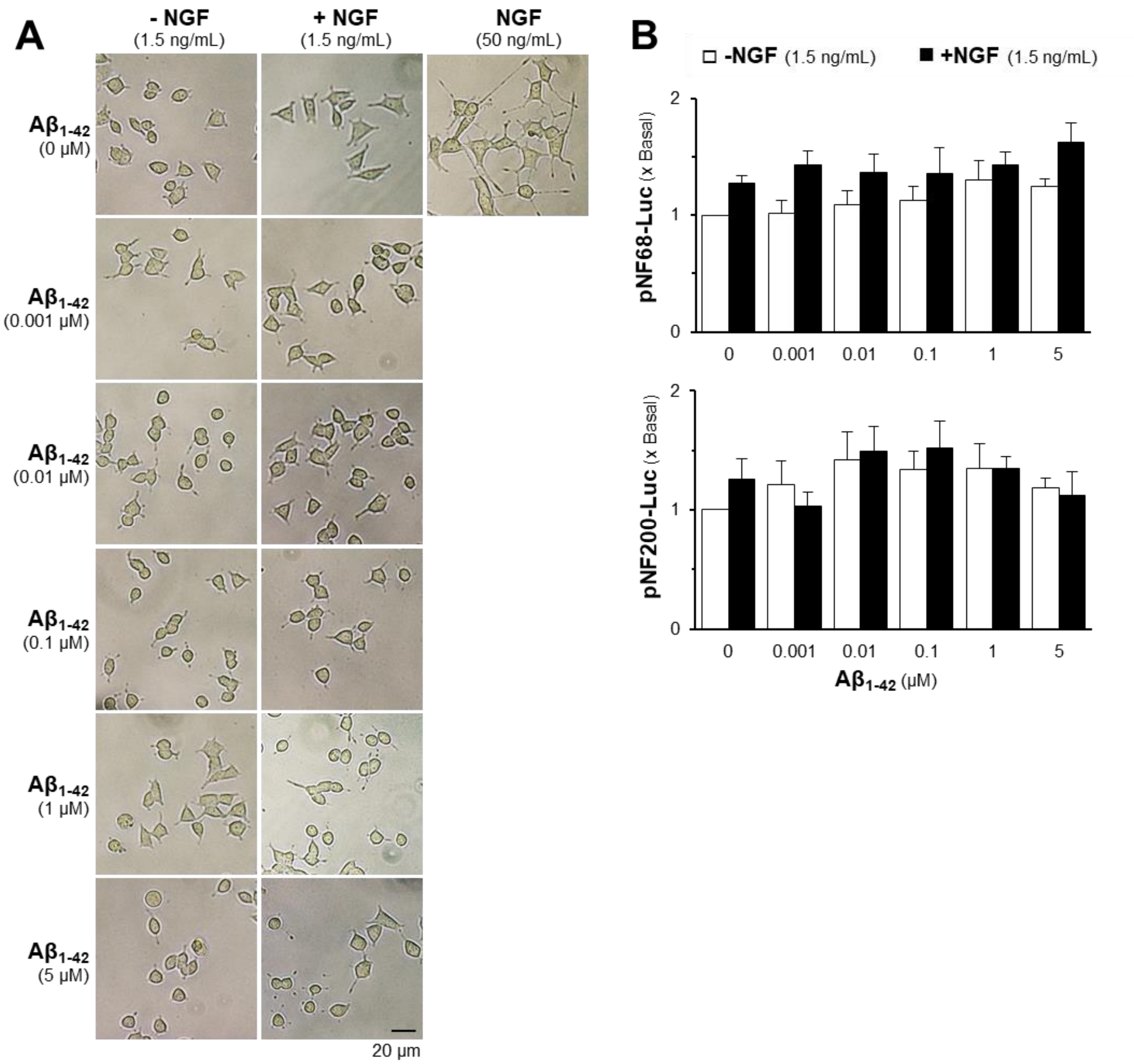

Supplementary Figure 5. Role of Aβ in inducing neurite outgrowth of PC12 cells. (A) The morphology of PC12 cells treated with various concentrations of Aβ, after 6-d aging with or without NGF (1.5 ng/mL) for 48 h was analyzed under a light microscope. NGF at a concentration of 50 ng/mL was used as a positive control. **(B)** Cells transfected with neurofilament promotor constructs pNF68-Luc and pNF200-Luc were treated with Aβ (6-d aging) alone or in conjugation with 1.5 ng/mL NGF for 24 h. Cell lysates were collected for determining luciferase activity. The luciferase activity is normalized to the amount of protein and represented as fold change, where mean ± SEM for *n* = 4.

Supplementary Table 1 HPLC analysis of *D. cochinchinensis* stemwood extracts.

Extracts	Yield (%)[a]	Identified compounds	Retention time (min)[b]	Peak area (mAU)[b]	Content (µg/mg)[b]
DCS_{EtOH90}	15.88 ± 0.19	Resveratrol	40.10 ± 0.09	2698.55 ± 360.28	13.01 ± 1.13
		Loureirin A	77.78 ± 0.07	634.10 ± 71.72	11.14 ± 0.64
		Loureirin B	78.85 ± 0.06	906.57 ± 121.61	11.08 ± 1.09
		Pterostilbene	84.54 ± 0.06	2898.48 ± 323.50	15.28 ± 0.56
DCS_{EtOH50}	16.70 ± 0.34	Resveratrol	40.16 ± 0.12	2912.62 ± 410.37	13.86 ± 1.20
		Loureirin A	77.85 ± 0.08	635.82 ± 91.03	11.12 ± 0.82
		Loureirin B	78.92 ± 0.08	914.02 ± 124.47	11.04 ± 0.80
		Pterostilbene	84.60 ± 0.07	2596.00 ± 335.08	14.31 ± 0.48
DCS_{water}	2.36 ± 0.08	Resveratrol	40.30 ± 0.18	1628.47 ± 393.51	7.71 ± 0.76
		Loureirin A	78.04 ± 0.19	84.10 ± 29.75	4.39 ± 0.58
		Loureirin B	79.30 ± 0.15	168.93 ± 30.05	0.84 ± 0.45
		Pterostilbene	84.85 ± 0.22	172.40 ± 32.98	1.27 ± 0.51

Ten mg of *D. cochinchinensis* extracts were dissolved with ethanol and analyzed by HPLC-DAD with a TC-C18 column (4.6 x 250 mm, 5 µm). The peak identities are indicated in Fig. 1.

[a] The yields in % per dried weight of herbal extract in Mean ± SEM, $n = 4$.

[b] The values are measured from HPLC analysis in Mean ± SEM, $n = 5$.
